# High-speed atomic force microscopy highlights new molecular mechanism of daptomycin action

**DOI:** 10.1038/s41467-020-19710-z

**Published:** 2020-12-09

**Authors:** Francesca Zuttion, Adai Colom, Stefan Matile, Denes Farago, Frédérique Pompeo, Janos Kokavecz, Anne Galinier, James Sturgis, Ignacio Casuso

**Affiliations:** 1 U1067 INSERM, Aix-Marseille Université, Marseille, France; 2 Biochemistry Department, University of Geneva, Geneva, Switzerland; 3 Organic Chemistry Department, University of Geneva, Geneva, Switzerland; 4 Department of Technical Informatics University of Szeged, Szeged, Hungary; 5 Laboratoire de Chimie Bactérienne (LCB), Institut de Microbiologie de la Méditerranée (IMM), CNRS, UMR 7283, Aix Marseille Université, Marseille, France; 6 Institute of Environmental Science and Engineering, University of Szeged, Szeged, Hungary; 7 LISM, UMR 7255, CNRS, Aix Marseille Université, Marseille, France

**Keywords:** Nanoscale biophysics, Applications of AFM

## Abstract

The increase in speed of the high-speed atomic force microscopy (HS-AFM) compared to that of the conventional AFM made possible the first-ever visualisation at the molecular-level of the activity of an antimicrobial peptide on a membrane. We investigated the medically prescribed but poorly understood lipopeptide Daptomycin under infection-like conditions (37 °C, bacterial lipid composition and antibiotic concentrations). We confirmed so far hypothetical models: Dap oligomerization and the existence of half pores. Moreover, we detected unknown molecular mechanisms: new mechanisms to form toroidal pores or to resist Dap action, and to unprecedently quantify the energy profile of interacting oligomers. Finally, the biological and medical relevance of the findings was ensured by a multi-scale multi-nativeness—from the molecule to the cell—correlation of molecular-level information from living bacteria (*Bacillus subtilis* strains) to liquid-suspended vesicles and supported-membranes using electron and optical microscopies and the lipid tension probe FliptR, where we found that the cells with a healthier state of their cell wall show smaller membrane deformations.

## Abstract (web summary)

High-speed atomic force imaging allows for the visualisation of molecular‐level activity in real-time. Here, the authors use HS-AFM to image the activity of an antimicrobial peptide on a membrane and are able to detect previously unknown molecular mechanisms behind its action.

## Introduction

The world is running out of antibiotics. In the last decades, antibiotic development has stagnated, while antibiotic resistance continues to rise. The European Centre for Disease Prevention and Control estimates that currently in Europe, 25,000 patients die per year due to antibiotic-resistant bacterial infections (https://ecdc.europa.eu).

Antimicrobial peptides (AMPs) have attracted considerable attention due to their wide spectrum of activity and the low degree of resistance^1,2^. Many AMPs function by targeting biological membranes, reducing the development of microbial resistance compared to intracellular targeting. Lipopeptides are a promising class of antibiotic drugs only investigated to a limited degree. They contain a hydrophilic peptide attached to a fatty acyl side chain that promotes insertion and clustering in the biological membranes^3^, and often incorporate non-ribosomal amino acids limiting their exposure to peptidase breakdown.

Lipopeptide action is largely determined by the length, saturation, and branching of the acyl chain^4,5^, there are often numerous homologues and isoforms, but the correlation of their structures with their precise actions is challenging; lipopeptide action is typically multifaceted, and numerous studies using multiple techniques have been deployed to understand their structure-function relationship^6,7^. There is an inherent complexity to predicting the consequences of small differences in the spatial arrangement of the amino acids in AMPs^8^. This uncertainty in the details of lipopeptide action hinders the pharmaceutical development of lipopeptides as antibiotics; the production of molecular candidates is costly and, as the chemical synthesis of common medically used lipopeptides is not viable at industrial scale^9,10^, the costs for pharmaceutical development are high. However it is now becoming clear that the toxicity of lipopeptides can be even lower than previously believed and additionally reduced by using new types of synthetical lipopeptides^11^, increasing their attractiveness for development.

Daptomycin (Dap) is the paradigm for the lipopeptide antibiotics incorporated to clinical use in the last forty years^12^. Therapeutically, Dap is used against Gram-positive (Gram+) bacterial infections including as a last resort antibiotic against multidrug-resistant enterococci and staphylococci^13,14^. Even if bacterial resistance to lipopeptides is low, in recent years, some strains have become resistant to Dap^15^. This antibiotic offers an excellent system for testing new approaches for the characterisation of the structure‐activity relationships of AMPs on membranes, with an aim to furthering development of this class of antibiotics and countering the spread of resistances.

What is known about Dap action? When a non-Dap-resistant Gram+ bacteria is exposed to Dap (in the presence of Ca^2+^), the plasma membrane of the bacteria depolarises in a slow process lasting several tens-of-minutes^16,17^. Currently, there is a knowledge-gap on the details of Dap activity. Up until now, it is not clear whether the observed depolarisation is the cause of bacterial mortality, or rather the depolarisation is the result of bacterial death caused by another mechanism^18,19^. To date, only the two initial steps of Dap action are well established. 1st, the anionic Dap molecule binds (in the presence of Ca^+2^) to the anionic phospholipid Phosphatidylglycerol (PG) found in the membranes of Gram+ bacteria. 2nd, after binding to the membrane, Dap is believed to undergo a structural transition inserting in the plasma membrane in a wedge-like configuration^20^, but assessment of the structural configuration of Dap in its membrane-bound state is still missing. The structural and dynamical pathways of the remaining stages of Dap action have not been clearly identified yet^21,22^.

The predominant hypothesis is that Dap antibacterial action is caused by toroidal pores that induce the depolarisation measured in bacteria^23–25^. The process of formation is believed to happen as follows: Dap monomers would oligomerize^26–29^; the large local positive curvature strain created in the outer membrane leaflet could result in dimples, also named half-toroidal pores^25^, that would locally reduce the thickness of the membrane through where some Dap molecules would translocate to the inner leaflet^25^ where they would oligomerize and in turn form similar dimples in the inner leaflet; finally, inner and outer dimples would fuse and produce the transmembrane toroidal pores^25,30^. Nevertheless, in the last six years, two other models of Dap action have been proposed, both based on optical microscopy (OM) observations. First, in 2014, the lipid extraction model was proposed in which Dap is suggested to cause ejection of material from the membrane^31^. Unfortunately, these observations were performed on membranes with a rather un-natural composition of 70% DOPC. Second, in 2016, in the membrane protein delocalisation model, it was proposed that Dap delocalises the glycotransferase enzyme MurG hampering bacterial cell wall formation^19^.

The wide range of techniques that have been used to measure AMPs activity (i.e., fluorescence resonance energy transfer (FRET), small angle neutron scattering (SANS), nuclear magnetic resonance (NMR), calorimetry, electrophysiology, X-Ray, or circular dichroism (CD)) provide little information on the oligomerization states which is an important piece of information missing in our understanding of the activity of the peptides^29^; as oligomerization controls the nature, dynamics and regularity of the membrane deformations. Atomic force microscope (AFM) and electrochemical scanning tunnelling microscopy (EC-STM) are unique in their capability of label-free high resolution imaging of biological molecules and their surroundings under physiological conditions, yet, in the literature, few studies address the effect of AMPs on supported lipid bilayers by AFM^32–44^ (Dap in particular was never studied), most are prior to 2011, few are the recent publications^42–44^. The published AFM data examines the evolution of the contours of membranes patches exposed to AMPs in solution. Never have the dynamics of the activity of AMPs been imaged at molecular resolution, for example oligomers could never be visualised due to their thermal diffusive motion. AFM and EC-STM molecular imaging was restricted to the static final states of the processes^32,39,42,43^, hence, overlooking the core of the AMPs molecular activity. Overall, the incapability to assert AMPs molecular activity hampers the straightforward correlation of the AFM data with the molecular, but averaged, information issued from the prevalent techniques of monitoring of AMPs molecular activity^36^. Alternatively, the all-atom molecular dynamics (MD) simulations of AMPs activity would provide the ultimate level of detail in the understanding of the mechanisms of action of AMPs; however the timescale of the pathways of action of AMPs on membranes is too long (several minutes^2,16,17^) and the numbers of peptide molecules involved in the processes excessive (as the ejections caused by Dap^31^) to be predicted by MD simulations; these are limited to the assessment of processes shorter than a few microseconds and only a handful of peptides^45^. Consequently, even if the currently available simulations provide us with valuable insights^46^, they cannot predict unknown pathways without prior knowledge from experiments—this is the case even for pore formation^46,47^—including the action of Dap^48,49^, as will be shown.

In the last decade, the advent of the high-speed atomic force microscope (HS-AFM)^50^ has achieved µs/pixel speeds. Using the HS-AFM, we could accomplish nanometre scale high resolution molecular imaging of the action of AMPs on membranes. For the first time it was possible to correlate the molecular picture of the activity of AMP provided by FRET, SANS, NMR, CD or electrophysiology techniques, with high resolution molecular imaging by AFM—non-averaged and label free—of the spatial membrane heterogeneity at local membrane environments with individual molecule resolution and dynamical information.

The imaging speed of scanning microscopies, like AFM, depends on the time of scan per one pixel. When particles move fast with respect the scanning speed of the microscope the particle traverses several pixels in the time the microscope scans one pixel, in this case it is not possible to define for each frame in which pixel the particle is located; the particle is not resolved in the image. To resolve the particle, it is necessary that the particle resides in the area of one pixel at least the time required for the microscope to scan one pixel. In biological membranes lipids and proteins diffuse driven by thermal fluctuations; their trajectories follow overlapping distributions of steps of variable sizes that depend on their interactions with their immediate membrane surroundings. The typical diffusion coefficients of lipids^51,52^ and small molecules as Dap^53^, are of order of few µm^2^/s, or in terms of instantaneous speeds (quotient of the mean free path and the step time) of the order of a few hundreds of µm/s^52^. Because (i) the imaging biomolecules at high resolution AFM requires a pixel lateral size smaller than 1 nm and (ii) the residence time of the membrane diffusing lipids and proteins in 1 nm^2^ is around 1–10 µs, it is necessary that the scan speed of the AFM is faster than 1–10 µs/pixel to attain visualisation of membrane diffusing lipids and small molecules. Prior AFM imaging of the action AMPs on membranes was performed at scan speeds from 200^44^ to 1000 µs/pixel^32–38^, thus too slow for visualising the molecular action of AMPs on membranes.

Biological membranes are extremely complex and dynamically partitioned into lateral micro-domains, a far from an ideal mixture of multiple proteins and lipids. The use of model lipid bilayers, artificially simplified systems, has been essential for our understanding of the roles of lipid, proteins and carbohydrates in drug membrane interactions^54,55^. Nevertheless, model membranes leave numerous factors unconsidered^56^: among others, the thousands of different lipid species present in biological membranes and the interactions with cytoskeletal components with active roles on membrane organisation are not present in the membrane models. Furthermore, in the case of AFM membrane imaging, the membranes are placed on flat supports, thus, additionally restricting the degrees of freedom for membrane deformations. Therefore, it is legitimate to ask ourselves the degree of relevancy of the data derived from supported model membranes, especially for the study of drug-bacterial membrane interactions. We have, in this article, sought to reinforce our AFM data, and address the relevance of our measurement, by correlating observations on several different systems progressively further from the native membrane. We have obtained information on living cells and non-supported model membranes by optical microscopy (OM), fixed cells and non-supported model membranes by electron microscopy (EM), and supported model membranes by HS-AFM. Moreover, besides structural assessment, we used the fluorescence dye FliptR to gain information on lipid packing; the fluorescence lifetime of FliptR depends linearly on membrane tension^57^. The images of deformations on supported membranes by AFM have been correlated with deformations and alterations of the lipid packing on cells and non-supported vesicles. Our multi-scale, multi-nativeness and multi-technique correlation of data lifts the significance of the ensemble of observations for the deciphering of the mechanism of action of Dap and other future drug developments.

Finally, the knowledge gap on Dap and other AMPs, molecular mechanisms can be partially ascribed at the uncertainties on the model membranes and conditions used in the experiments, we list: (i) The *fluorescence labels*, commonly used, can modify Dap function; as shown for BODIPY-labelled^58^, perylene-labelled^28^ and NBD-labelled Dap^49^, (ii) *Room temperature* which is the temperature most commonly used in Dap studies, rather than the physiological temperature (37 °C) pertinent to antimicrobial use, can strongly modify membrane properties and thus alter Dap action^21,59,60^. Finally, (iii) The model membrane *lipid composition* can modify membrane properties and membrane-Dap interactions: (iii.a) The *lipid head group*, phosphatidylcholine (PC) has been commonly used in model membranes, but PC is not found in Gram+ bacteria and can alter Dap action^18^, (iii.b) The *acyl chain*, dimyristoyl (DM, 14:0–14:0) lipids were the most common used acyl chain. However, it is known that Dap permeabilizes lipid bilayers with DM acyl chains, but not membranes with the more fluid palmitoyl-oleoyl (PO, 16:0–18:1) or dioleoyl (DO, 18:1–18:1) acyl chains^61^, what is more, the DO acyl chains are the most common acyl chain in *Staphylococcus aureus*, and infection by this bacteria is commonly treated by Dap administration^53^. In our experiments, to minimise the uncertainty related to the pertinence of the observation conditions, we mimicked as close as possible the bacterial infection conditions.

As mentioned above, since bacterial resistance to Dap is unfortunately increasing^15^, understanding how bacteria resist Dap is increasingly relevant. The Dap resistant bacterial strains present amended cell walls and/or plasma membranes structures^62^. It is known that at the plasma membrane of the Dap-resistant strain of *Enterococcus faecalis*, a bacterial infection commonly treated with Dap, the content of cardiolipin (CL) is higher than in membranes of the non-Dap-resistant strains^63,64^. However, the mechanisms by which CL inhibits Dap action remain unclear^30^. In model membrane assays—regrettably containing >40% of the non-bacterial PC lipid—the addition of CL at 10–20% molar to the membrane inhibited the permeabilization of the membrane by Dap^25,60^. Herein, we provide novel insight on CL-Dap interactions by HS-AFM imaging correlated to electron and optical microscopies on PG membranes.

In this work, the function of Daptomycin, the paradigm of the antimicrobial lipopeptide molecules, whose multifaceted mechanisms remains for the mostly part unknown is assessed by a framework of assays. These assays significantly include the single-molecule visualisation of the activity of lipopeptides on membranes. The manuscript starts by the higher nativeness measurements in living bacteria, and progressively decreases the nativeness, approaching supported membranes and inversely increases the resolution. All measurements mimicked the infection conditions: 37 °C, bacterial lipid composition and antibiotic concentrations. We also mimicked bacterial resistance conditions by increasing the CL-content in the membrane. We confirm several, previously, hypothetical models, and detect several unknown molecular mechanisms. Moreover, we provide quantitative information on the Dap oligomers and their interactions.

## Results

### Dap action on bacteria and non-supported vesicles

Three different strains of the Gram+ bacteria *Bacillus (B.) subtilis* were exposed to Dap and imaged by OM (using the membrane specific dye FM1–43) and scanning electron microscopy (SEM). The strains studied were the wild-type (WT)^65^ and two cell strains with defective cell walls; 1st, a mutant lacking *ugtP*^66^; UgtP is an enzyme involved in the synthesis of glycolipids and the anchoring of lipoteichoic acids that is required for the cell wall formation of Gram+ bacteria and that was suggested to be inhibited in the cells exposed to Dap^67^; 2nd, a mutant lacking the actin-like protein MreB^68^; this cytoskeletal protein is involved in the patterning of the bacterial membrane, among others defining the location of the regions of increase fluidity^69^ where Dap preferentially inserts^19^.

The lowest concentration of Dap that inhibited the visible growth of *B. subtilis* in overnight incubation was measured (3.1 µM for all the strains), in agreement with published values for *B. subtilis*^19^ and other Gram+ bacteria^70^. For all the rest of this study, two concentrations of Dap were used: (1) A low concentration of 0.6 µM, below the minimal inhibitory concentration (MIC). and (2) a high concentration of 20 µM, above the MIC. Importantly, these Dap concentrations are in the range of that found in the serum of patients during Dap treatment^71^. At the lower concentration depolarisation of the *B. subtilis* cells has been measured using a membrane potential-sensitive fluorophore^19^. It should be noted that for a component partitioning into the membrane the concentration in the aqueous phase is perhaps not the most pertinent.

**Fig. 1:**
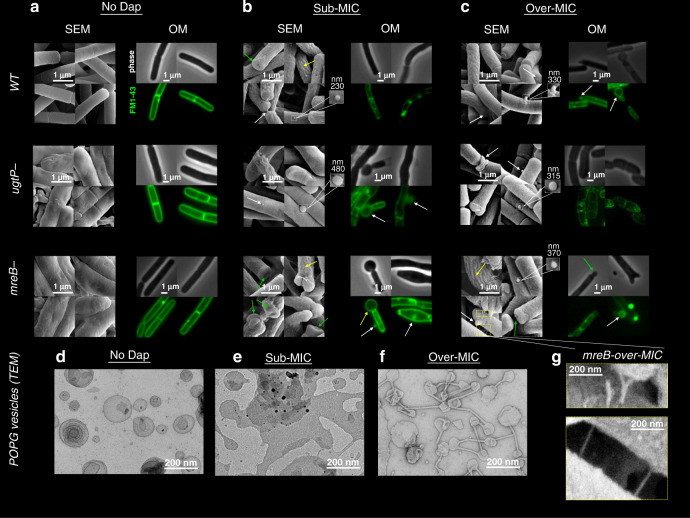
Dap action on *B. subtilis* strains (WT, Δ*mreB* and Δ*ugtP* strains) and on POPG vesicles by EM and OM. **a** In the absence of Dap, the Δ*mreB* and Δ*ugtP* mutant strains display abnormal surfaces visible by SEM, whereas the OM does not display such disparities. **b** Exposed for 15 min to sub-MIC Dap, SEM displays in the WT and Δ*mreB* strains, but not in the Δ*ugtP* strain, the creasing of the bacterial cell wall surface (yellow arrows). In all the strains, we observe pronounced deformations at the cell poles (green arrow) and submicron protuberances emerging, seemly of lipidic nature given its spherical shape (white arrow and insets). In contrast, the OM displays bulbous micron-sized swellings, primarily coming out of the bacterial poles in the two mutants, but not in the WT cells. Thus, the cell wall delimits the Dap-induced morphological alterations. **c** Exposed for 15 min to over-MIC, the OM also displays bulbous micron-sized swellings in the WT *B. subtilis*. All the rest of deformations observed at sub-MIC are found also at over-MIC in all the strains. **d**–**f** TEM of POPG vesicles exposed to Dap: In the absence of Dap, the POPG vesicles display spherical shape; at sub-MIC, a number of tubulations emerge from, and interconnect, the vesicles (black arrows); at over-MIC, the tubulations are omnipresent interconnecting the vesicles and the surface of the vesicles becomes creased. **g** Detail of the tubulations that appear in the Δ*mreB* cells at over-MIC visualised by SEM. Their size, shape, and interconnective character, is comparable to those observed in POPG vesicles shown in **e** and **f**. These tubules are only noticed with Δ*mreB* mutant; thus, they are not created by the SEM sample preparation.

The three strains of *B. subtilis* were exposed to sub-MIC and over-MIC Dap for 15 min in physiological buffer with ∼1.25 mM Ca^2+^ at 37 °C. All the strains displayed significant deformations observed by OM (by phase contrast and FM1-43 membrane labelling) and SEM. Notably: submicron sized protuberances emerged from the bacterial wall, these are probably lipidic given their spherical shape and staining with FM1-43 (Fig. 1b, c, white arrows in the OM imaged plus the insets in the SEM images); the roughening of cell wall surface, in the WT and the *mreB* mutant strains at sub-MIC, and in all the strains at over-MIC (Fig. 1b, c, SEM images, yellow arrows); and, lastly, in all the bacterial strains, some cells developed large spherical micron sized vesicles at their poles (Fig. 1b, c, green arrows), reminiscent of the Dap-induced lipid extraction effect on model membranes^31^ (Fig. 1b, c, OM images, green arrows). The fact that at sub-MIC, the lipid extraction takes place only in the cell wall defective strains, not in the WT strain (confirmed by Müller et al.^19^) brings to light that a ‘healthier’ cell wall restricts the growth of the Dap-induced morphological alterations of the bacterial membrane. This could result from slowed Dap penetration through the native cell wall and/or increased resistance to membrane deformations. The comparable MIC value of the three strains would suggest the arrival of Dap to the membrane is not changed among strains.

**Fig. 2:**
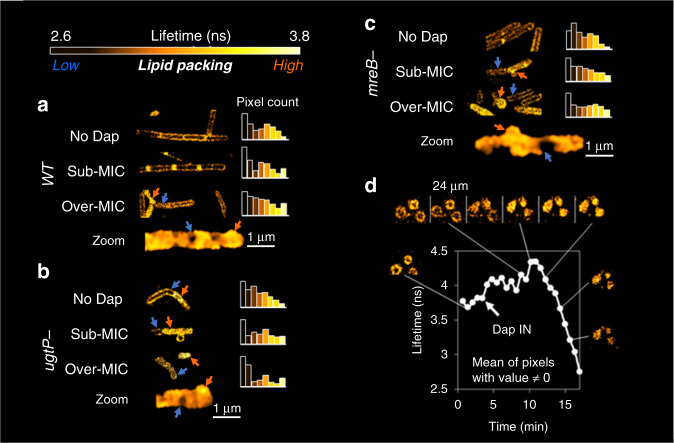
Dap action on *B. subtilis* strains (WT, Δ*mreB* and Δ*ugtP* strains) and POPG vesicles by the FliptR lipid packing probe. FliptR signal: lower lifetimes correlate to lower lipid packings and vice versa. **a** FliptR image on WT cells: in the absence of Dap, all the cells keep the same distribution, the bacterial poles display higher packing levels than the bacterial sides, a bimodal distribution of lipid packing values appears; at sub-MIC, at the poles, lipid packing level rises; at over-MIC, an inter-cell heterogeneity appears, certain cells show high lipid packing disseminated over the full cell (orange arrow) while others maintain their previous packing distribution (blue arrow), the distribution of packing stretches and a little peak at higher levels of packing becomes apparent. At higher magnification (zoom) also heterogeneity at the intra-cell level becomes evident, zones of high (orange arrow) and low (blue arrow) lipid packing disseminate inside the cell, in agreement with previous observations^19^. It is observed that the deformations protruding from the cells present higher levels of lipid packing than the zones that do not protrude. **b**, **c** For the Δ*mreB* and Δ*ugtP* mutants, FliptR signals are akin to WT strain. Yet, the inter-cell heterogeneity appears at lower Dap concentration, sub-MIC instead of over-MIC, supporting that ‘healthier’ bacterial cell wall limits the deformations induced by Dap. **d** Tracking of the progression of the lipid packing on POPG vesicles exposed to over-MIC Dap by FliptR. Dap is injected at the ‘Dap IN’ label and a two-step process takes place. First the vesicles rigidify, then they collapse and the lipid packing is drastically reduced to values below the initial ones. This two-step process could explain the two zones of low and high lipid packing identified in the bacteria cells exposed to Dap.

To gain insight at the molecular-level, we used the fluorescence dye FliptR, an indicator of the membrane lipid packing^57^. In the absence of Dap, the intra-cellular distribution of the lipid packing is similar for all the bacterial cells, with a bimodal distribution showing tighter lipid acyl chain packing at the poles and looser packing at the sides. In the WT cells, low concentrations of Dap seem to slightly increase packing at the poles. However, higher Dap concentrations increase the heterogeneity in the distribution: We observe tight lipid packing over the totality of some cells (Fig. 2a, WT, over-MIC, orange arrow), while others maintain the loose packing found in the absence of Dap (Fig. 2a, WT, over-MIC, blue arrow); as a result the packing distribution is no longer bimodal but stretched and with a little peak at higher levels of packing. Furthermore, not only there is a heterogeneity between cells but the intra-cellular heterogeneity also increases. The Dap induced deformations of the membranes are heterogeneous and result in zones of high and low lipid packing with higher levels of lipid packing at the protrusions (Fig. 2a, WT, over-MIC, zoom, orange and blue arrows). Similar observations were made by Müller et al. in 2016 using the hydration of the Laurdan membrane probe for membrane fluidity assessment^19^, more dependent on the lipid composition than the FliptR lipid packing assessment (moreover, the intrinsic kynurenine fluorescence of Dap could have caused data misinterpretation, as it was not corrected^19^). In line with the cellular morphological observations, the lipid packing observations show that on the mutant strains smaller concentrations of Dap induce the same effects as higher concentrations of Dap on the WT (Fig. 2b, c); the finding reinforces the idea that a normal cell wall is probable capable of limiting Dap-induced deformations.

To gain further insight, vesicles with lipid composition similar to that of the pathogens *Clostridium difficile*^72^ and *S. aureus*—both medically treated with Dap—1-palmitoyl-2-oleoyl, (PO, 16:0-18:1 acyl chains) PG (POPG) were characterised with same set of measurements as the bacteria. We avoided mixing the anionic POPG with non-bacterial zwitterionic PC lipid as many previous authors have^18,21,24–27,31,33,35,37,38,44,48,49,60,61^. TEM shows that, unsurprisingly and as in bacteria, Dap deformations are more important at higher than at lower Dap concentrations (Fig. 1d–f). We observed that the predominant type of deformation was the formation of membrane tubules that resembled, in shape and dimensions, those that emerge from the surface of *mreB*-mutant cells at high Dap concentrations (Fig. 1g). Significantly, the fact that similar tubules were noticed in one out of the three *B. subtilis* strains shows that they are not artefacts from the SEM sample preparation procedures. The observation of such tubules could correlate with the lipid extraction model derived from similar observations of (7:3) DOPC:DOPG vesicles^31^. By tracking the development of the lipid packing on POPG vesicles following exposure to Dap using FliptR (Fig. 2d), we observed a two-step process: after an initial stage of increase in packing, there is a dramatic reduction in lipid packing, correlating to intense deformation of the vesicles. This two-step process, with both reduced and increased lipid packing, is consistent with the heterogeneity of zones identified in the bacteria exposed to Dap; the POPG vesicles exposed to Dap mimic the morphological and the lipid packing effects observed in bacteria, thus supporting the use POPG supported model membrane for the assessment of the activity of Dap by AFM.

### HS-AFM on supported POPG bilayers

In order to observe at the molecular-level the effects of Dap on lipid bilayers we imaged supported POPG bilayers by HS-AFM that featured an imaging rate of about ten frames per second on areas of hundreds of nanometres square at sub-nm resolution.

**Fig. 3:**
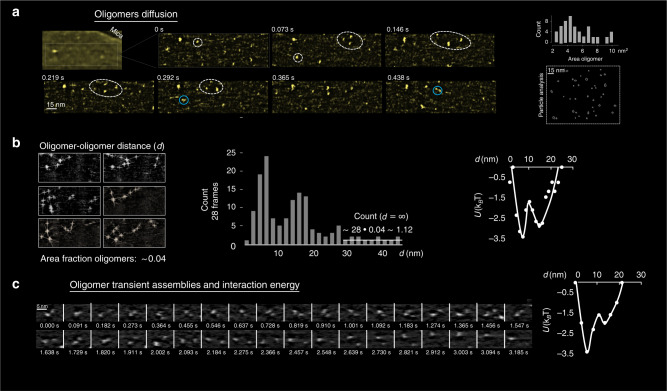
Sub-MIC Dap on POPG at 37 °C. First minutes. Initial stages. **a** Oligomers of Dap in diffusion on a membrane (slashed circles). The upper left image shows the membrane and the underlying mica substrate (colour scale: 4 nm). The rest of the images are close-ups on top of the membrane (colour scale: 2 nm): The oligomers formed temporary ensembles (slashed ellipses), but they did not form permanent clusters. Movie details: frame rate 74 ms; full image size 180 nm × 60 nm; full-frame size 256 × 80 pixels. **a**, right, The particle analysis (right bottom, one of the frames analysed; right top, histogram) shows a spread in the area of the oligomers from 2 to 10 nm^2^, a peak value of 4.3 nm^2^, and a mean and standard deviation of 5.4 ± 3.2 nm^2^; which corresponds to a stoichiometric variability maximal of 1 to 6 monomers, and mean and standard deviation of 3.6 ± 2.1 monomers. The oligomers experience mutual attraction at short distances. **b**, left, Oligomer-oligomer energy profile obtained from the spatial distribution of oligomer-oligomer distances. The area fraction of the oligomers on the membrane is of ~0.04. **b**, centre, Minima are found at distances between oligomers of ~7 and ~16 nm. The count at far distance (*count* ∞) can be calculated to be ~1.12 (area fraction occupied by the oligomers (0.04; ~4% of the total membrane area) multiplied by the number of frames counted (*n* = 28), horizontal white line at *d* > 30 nm) in agreement with the count at *d* > 30 nm. **b**, right, Equilibrium Boltzmann distribution energy profile derived from U(*d*) = −k_b_Tln[*count* (*d*)/*count* ∞]; the depthless of the energy wells is estimated to be of −3.4 k_B_T at *d* = 7 nm and −2.8 k_B_T at *d* = 16 nm. **c** These 32 frames (2.821 s) show a pair of oligomers in interaction. **c**, right, From the count of *d*, the energy profile features one global (*d* = 7 nm) and one local (*d* = 16 nm) minima. Because the oligomer-oligomer interaction is ergodic and, as expected, the interaction profile in space is similar to interaction profile in time. This allows to derive the energy values of the interaction profile in time from the in space results.

The HS-AFM allowed us to observe the development of the dynamics of interaction at the molecular-level over several hours. Over the course of the experiment many different structures were observed forming and then disappearing giving rise to new structures. At the first stages, measured at low Dap concentration and 37 °C, it was possible to observe small particles occupying an area fraction of ~4% of the total surface diffusing rapidly on the POPG (Fig. 3a and Supplementary movie 1). These particles diffused, as expected^52^, at speeds of 300 ± 200 µm/s, or in terms of diffusion coefficient a few µm^2^/s, which is consistent with the particles being Dap oligomers. Understanding the oligomerization states is critical to understand the function-structure relations of AMPs^73^. The images confirm that the HS-AFM imaging enables molecular-level visualisation of the transients of AMP oligomers in diffusion on membranes label-free, hence without financial cost and the incertitude related to the labelling of AMPs^28,49,58^ that hampers pharmaceutical development. In these initial stages of action, the oligomers created temporary ensembles (Fig. 3a, slashed ellipses), but they did not form permanent clusters. Nevertheless, we noticed that the Dap oligomers form transiently stable assemblies: mainly pairs of oligomers (Fig. 3a light blue circles; Fig. 3c). These transient assemblies of oligomers diffused and rotated and re-separated (Fig. 3c, last frames). Their presence indicates that at sufficiently short distances there is a net attractive force between the Dap oligomers. Further detail was obtained by following two interacting oligomers, we obtained a movie of 36 frames where the oligomers rattle in mutual attraction after correcting for their lateral diffusion (Fig. 3c), we measured the probability distribution (in time) with respect to the centre-to-centre distance (*d*) and derived the energy profile U(*d*) considering an equilibrium Boltzmann distribution^74^ (Fig. 3c, right), moreover using the spatial distribution of the ensemble of oligomers (which is equivalent by ergodicity to time probability distribution) we further evidenced the oligomer-oligomer attraction and the shape of U(*d*). We could quantify that the U(*d*) is attractive for *d* < 20 nm with a global minimum of energy of −3.4 k_B_T at *d* = 7 nm, and a local minimum of −2.8 k_B_T at *d* = 16 nm; such multi-minima interaction energy profile could cause bi-stable behaviours in the membrane deformations induced by Dap. To date, the membrane-mediated lipopeptide oligomer-oligomer interactions were not studied neither theoretically nor numerically, only the formation of the individual oligomers was so far simulated^75^. Nevertheless, there have been experimental reports of the aggregation of Dap oligomers; by Förster resonance energy transfer (FRET), where it was found that the signal of the oligomer-oligomer interaction exceeds pure random interactions^27^; and by OM on living cells, where clusters of Bodipy-labelled Dap molecules were identified on *B. subtilis*^58^. Curiously, the attractive interaction detected for the Dap oligomer contrasts with the repulsive interaction expected for membrane-bound circular and small curvature-inducers^76^; besides the lack of theory, the fatty acyl side chain of Dap is probable a crucial point to explain the observed attraction. Structurally, the oligomers show a diversity of sizes, the analysis indicates (Fig. 3a, right) a range of areas from 2 to 10 nm^2^, with a median of 4.3 nm^2^, and a mean and standard deviation of 5.4 ± 3.2 nm^2^. In view of the diameter of the Dap hydrophilic peptide of 1.4 nm, and its area of 1.5 nm^2^, the Dap oligomers stoichiometry ranges 1 to 6 monomers, with a mean and standard deviation of 3.6 ± 2.1 monomers. This range compares well, although with higher spread, with a previous FRET estimation (even if that calculation supposes homogeneous stoichiometry) of the Dap oligomer containing 6 to 7 monomers^27^, and a more recent estimation of the stoichiometry of Dap oligomers using electrophysiology renders a spread closer to our findings^77^.

In summary, during the first minutes of exposure, Dap is able to insert the exposed lipid leaflet and once inserted, the monomers diffuse rapidly. The HS-AFM allowed us to confirm the formation of oligomers without the uncertainty^26–28^ linked to label-based approaches. Here, we observed a slightly wider distribution than previous measurements^27^ of the Dap stoichiometry by FRET that ranged from 1 to 6 monomers, but close to the estimations by electrophysiology^77^. In addition, the extent of our size distribution could be partially issued from the uncertainties derived from process of AFM imaging of the fast moving (300 ± 200 µm/s) oligomers. Significantly, our data points to an oligomer-oligomer attraction for centre-to-centre distance below 20 nm in which a partially bi-stable energy well is present: circumstance that could cause the emergence particular supramolecular organisations on the membrane that could be relevant for Dap activity.

**Fig. 4:**
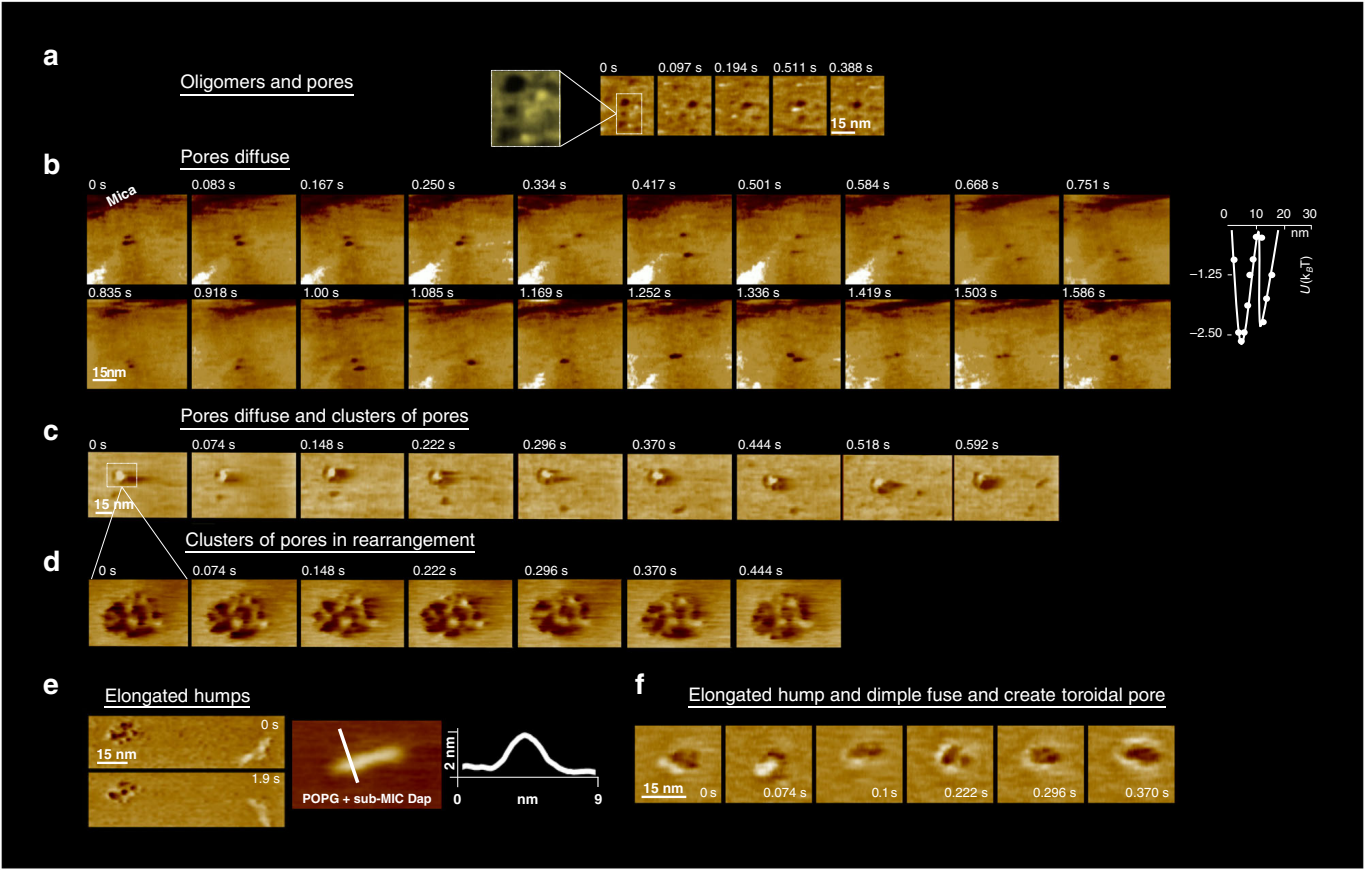
Sub-MIC Dap on POPG at 37 °C. Tens of minutes. Intermediate stages **a** A new structure appeared: dimples, zones of thinner membrane thickness, whose diameter was in the range 7 ± 2 nm. Most dimples diffuse, but some remained static (colour scale: 3 nm). Movie details: frame rate 97 ms; zoom of a full image of 150 nm × 90 nm and 256 × 160 pixels. **b** The dimple diffusion consisted of swinging trajectories, implying membrane-mediated dimple-dimple attraction (colour scale: 3 nm). **b**, right, Energy profile of the interaction of the dimples obtained derived from 120 centre-to-centre distance measurements that contains as the oligomers two energy minima. Movie details: frame rate 83 ms; full image of 150 nm × 150 nm and 256 × 256 pixels. **c** In some membrane zones, clusters of dimples, reminiscent of cubic phases, developed (colour scale: 4 nm). Movie details: frame rate 74 ms; full image of 90 nm × 60 nm and 256 × 160 pixels. **d** The clusters of dimples were moderately dynamical in time, with moderate internal rearrangements (colour scale: 4 nm). Movie details: frame rate 74 ms; full image of 25 nm × 16 nm and 256 × 160 pixels. **e** The other deformation found was elongated-humps on top of the POPG membrane. **e**, left, An elongated-hump in the proximity of a cluster of dimples (colour scale: 4 nm). **e**, right, A close-up and a profile of an elongated-hump. Additional images of elongated-humps on Supplementary Fig. 1. Movie details: frame rate 479 ms; zoom of full image of 250 nm × 200 nm and 300 × 256 pixels. **f** It was observed that the dimples and the elongated-humps fused and gave yield to pores of toroidal structure where a protruding ring surrounds the pore (colour scale: 4 nm). Movie details: frame rate 74 ms; full image of 40 nm × 40 nm and 256 × 160 pixels.

After a few tens of minutes of exposure, the observable structures change. Our imaging observed ‘dimples’, circular zones of decreased membrane thickness, as proposed by Zhang et al.^25^, diffusing on the membrane along with the oligomers (Fig. 4a) with an instantaneous speed (0.7 ± 0.5 µm/s, Fig. 4b, c) that is roughly six times slower than that of the Dap oligomers; still the AFM speeds from 6 to 60 µs/pixel that the dimples required for their imaging were too fast for the conventional AFM rates of 200^44^ to 1000^32–38^ µs/pixel. The dimples had slightly variable diameters of 7 ± 2 nm and depths of 0.9 ± 0.3 nm (88 measurements) suggesting little variations in the number of molecules forming them. From the swinging trajectories of diffusing dimples, it was clear that the membrane tension they generated resulted in attractive forces (Fig. 4b and Supplementary movie 2). It was also clear that the dimples of the same leaflet can fuse and give yield to dimples of larger size in that same leaflet (Fig. 4b, last frame); not be confused with the fusion of dimples from opposite membrane leaflets that is proposed in the existing model of Dap pore formation^25^. In more detail, their energy profile (Fig. 4b right, from *n* = 120 dimple-dimple distance measurements) shows a similar pattern that the oligomers, two minima appear at *d* ~ 5 nm, where the dimples are in a side-by-side contact, and at *d* ~ 13 nm, where they interact by the stress field they generate in the bilayer. In agreement with the attraction between dimples, we were able to observe clusters of dimples (Fig. 4c), not previously described in the literature, slightly reminiscent of cubic phases, located statically on the membrane, although with internal dynamic rearrangements on the time scale of several seconds (Fig. 4d).

In addition to the oligomers and dimples, at slightly longer times after exposure to Dap, another type of deformation, previously unreported in the literature, that we named ‘elongated-humps’ formed on top of the POPG bilayer surface (Fig. 4e and Supplementary Fig. 1), and presented rather uniform widths and heights, with 4 nm and 2 nm, respectively, but variable lengths ranging from several to tens of nanometres.

Significantly, due to the fast imaging speed of the HS-AFM, we could determine the process of fusion between the elongated-humps and the dimples that ended in the formation of toroidal-shaped pores (Fig. 4, 2f). In this process, never observed nor proposed for Dap molecular activity, an elongated-hump embraces the dimple forming a ring protruding 2 nm above the membrane, which then develops into a toroidal membrane pore.

**Fig. 5:**
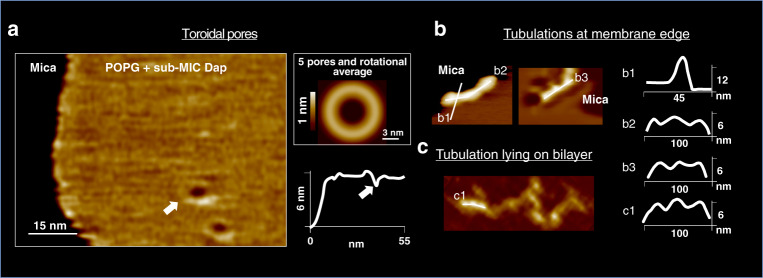
Sub-MIC Dap on POPG at 37 °C. Hours. Final stages. **a** Toroidal pores of similar topography than those formed from the fusion of a hump and a dimple formed. **a**, left, Two toroidal pores in close to the membrane border, the underlying mica support is indicated (colour scale: 3 nm). **a**, right, An average, including rotational average, of 5 toroidal pores. Toroidal pores did not diffuse, in sharp contrast to the diffusive dimples. The AFM tip radius was too large for the height profile to assess the depth of the toroidal pores, and thus, if they traversed the membrane (white arrow in height profile). Supplementary Fig. 2 provides further details the AFM topographic profiles of the toroidal pores. **b** Tubulations formed, mostly at the edge of the POPG membranes. The tubulations were nearly rectilinear and displayed regions of larger and thinner cross sections that periodically distributed with a ~34 nm period, which is symptomatic of a two components composition in the tubules, Dap and POPG, at the thinner and larger cross section regions, respectively. Height profiles across and along the tubules are shown. **c** More rarely, tubules were found lying on the POPG bilayer, some sections, the more rectilinear ones (k1), present periodicity in their topography, other, the less rectilinear, do not show topographic periodicity.

To later stages of Dap action, after several hours, the objects visible in the membrane had evolved further. Toroidally shaped pores, of similar shape than the pores formed by the fusion of humps and dimples, appeared on the POPG membrane (Fig. 5a), the AFM visualisation reinforces the toroidal pore model of Dap action^25,30^, and the timescale of formation of the pores relates well to the Dap-induced depolarisation process observed on bacteria that lasts several tens-of-minutes^16,17^. The toroidal pores were differentiated from other structures by their larger diameter, a protruding external ring, and their lack of mobility that contrasted strongly with the dynamic dimples. These differences suggest that the toroidal pores could traverse the membrane, as proposed by the toroidal pore model. It would be possible that the toroidal pores may not diffuse because they interact with the underlying mica substrate. Unfortunately, the HS-AFM probe tip is too large to fit into the toroidal pores, and consequently we cannot determine whether the toroidal pores traverse the membrane or not (Fig. 5a and Supplementary Fig. 2). The geometry of the pores often deviated from ideal toroidal shapes, often the pore showed an elliptical or incomplete couronne; in an ensemble of 16 pores, the couronnes of the pores range in their peak-to-peak distance between 7 and 12 nm (Supplementary Fig. 2). Another structure that formed at the long times were tubulations with diameters between ~8 and ~12 nm, these were mostly observed at the membrane edges (Fig. 4b), and rarely lying on the POPG bilayer (Fig. 4c). These structures may be related to the tubules visualised on POPG vesicles and *mreB* mutant cells (Fig. 1f, g). They are probably also related to the elongated-humps observed. Significantly, the tubulations where not uniform but displayed regions of thicker and thinner cross-section with a periodicity of ~34 nm (Fig. 5b): only the most rectilinear sections of the tubules showed periodicity, at the membrane edges the tubules were highly rectilinear, on the tubules lying on the bilayer the curved sections did not show the periodicity that arose in the most rectilinear sections (Fig. 2b, c). This periodic structure, alternating positive and negative curvature, indicates in the present case that the tubules are composed of more than one species, presumably POPG and Dap; since POPG, a cylindrical lipid, forms lamellar phases, while Dap alone forms spherical micelles^78^. Provided their respective curvatures, we reason that the thinner and thicker regions of the tubules are enriched in Dap and POPG, respectively. In sum, our results on living bacteria and membranes are consistent with the lipid extraction model^31^ that was before witnessed on membranes of 70% content of PC a lipid not found in Gram+ bacteria that may alter Dap activity^18^.

It should be remembered that the geometry of this experiment means that, even though the concentration of Dap is low, the Dap to lipid ratio is rather high, as the lipids are confined to the mica surface. Thus, a considerable amount of Dap can partition into the supported membrane. A further peculiarity of the geometry is that the supported bilayer is attached to the mica surface and thus the leaflet in contact with the support is inaccessible for Dap insertion. Membrane attachment also is likely to hider the deformations of the lower leaflet necessary for the formation of toroidal pores. Nevertheless, we observed a number of structures probably pertinent for Dap action: The fast diffusing (300 ± 200 µm/s) small oligomeric clusters of the first minutes developed into slower (0.7 ± 0.5 µm/s) diffusing dimples, elongated-humps on top of the membranes, and static clusters of dimples, that ultimately gave rise to pores, cubic phases and tubules. Our visualisations are consistent with both the toroidal pore and lipid extraction models.

**Fig. 6:**
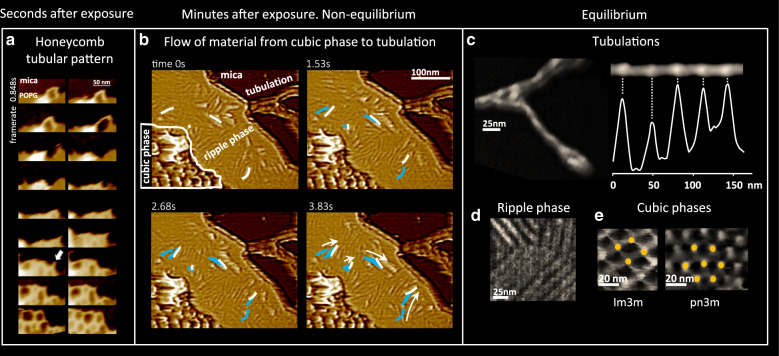
Over-MIC Dap on POPG at 37 °C. **a** The first seconds after exposure to over-MIC Dap, at the POPG membrane border tubules of diameter of ~15 nm branch in hexagonal directions (arrow) and create in seconds a tubular honeycomb structure. Movie details: frame rate 848 ms; zoom of a full image of 150 nm × 90 nm and 256 × 160 pixels (colour scale: 16 nm). **b** Minutes after exposure, the POPG membrane showed an heterogenous and dynamic environment where different regions of the membrane showed different Dap-induced deformations and where material flowed from region to region (low passed filtered images to highlight the contrast are shown). The flow of material, observable on a zone of ripple phase of 15 nm pitch (please note that the white and blue lines and the arrows indicate the direction of displacement of the ripples), indicative of a non-equilibrium situation, went from the cubic phase type Im3m of 25 nm of pitch to the tubulation that emerged from the border of the POPG patch. Movie details: frame rate 1530 ms; full image of 390 nm × 350 nm and 256 × 256 pixels. **c** Once the membrane exposed reached equilibrium (1 h after exposure), higher resolution imaging of the effects of over-MIC Dap exposure was possible. The tubulations (of Fig. 3b) were observed to be of periodic shape with zones of thicker and thinner diameters distributed with a 32 ± 4 nm period. Compared to the tubulations found at sub-MIC, the zones of thinner diameter were appreciably longer, given the higher content on Dap at over-MIC than at sub-MIC, the zones of thinner diameter were associated with zones enriched in Dap and, inversely, the zones of thicker diameter with zones enriched in POPG. **d** At equilibrium, also the ripple phase could be imaged in more detail, as well as **e** Im3m and Pn3m cubic phases.

The picture was rather different at higher Dap concentration. The first seconds after exposure, on the edge of the bilayer, tubules formed and branched into and hexagonal honeycomb structures of variable and time-changing unit cell size that evolved into a smaller and more uniform unit cells of ~55 nm (Fig. 6a). After the first minutes after exposure, the membrane was fully covered with a heterogeneous ensemble of deformations. However, no dimples or toroidal pores, common at lower concentrations, were observed. The deformations were very dynamic as the system reconfigured. The structures were clearly in a non-equilibrium situation and material flowed across the membrane (Fig. 6b and Supplementary movie 3). The flow of material streamed across a pronounced structural heterogeneity: the flow seemed to originate at a structure similar to an Im3m cubic phase (25 nm of pitch) reminiscent of the aggregations of pores at lower concentration and the hexagonal tubular honeycomb, and end at a tubulation located at the edge of the membrane patch, the flow traversed a rippled membrane (15 nm of pitch) where the ripples, slightly reminiscent of the membrane humps, moved and indicated the sense of the flow (Fig. 6b, white and blue lines); even if only at the rippled membrane could the flow of material be assessed by the HS-AFM, provided the conservation of the flux continuity, even if not seen by HS-AFM, material should flow too across the cubic phase domain and the tubulation.

After 1 h, the dynamic flow came to a stop, equilibrium was reached, which enabled us to achieve higher resolution AFM imaging. We imaged tubules that were similar to those observed when the membrane was exposed to sub-MIC Dap concentrations (Fig. 5b): also they presented periodically distributed regions with smaller and thicker cross sections, their periodicity was of ~32 nm, hence, similar to the ~34 nm period measured at sub-MIC (Fig. 6c), indicating that the ratio Dap:POPG inside the tubules is relatively invariant with respect the concentration of Dap in solution. The equilibrium staticity also facilitated us a better imaging of the ripple phase, as well as the Im3m and pn3m cubic phases (Fig. 6d, e). The spacing of the ripple phase regions was evaluated at 13.1 ± 0.9 nm (Supplementary Fig. 3), and of the Pn3m and Im3m cubic phases at 11.2 nm and 16.7 nm, respectively, the sides of the hexagon and a square patterns (Supplementary Fig. 4).

The observations at over-MIC are less easy to relate to the physiological action of Dap. However, they highlight a number of important points such as the relaxation of an out of equilibrium situation through the formation of Dap-POPG tubules. The situation at over-MIC, in which Dap binding to the membrane is much faster, results rapidly in a very rich and congested membrane with little space available for the formation of dimples or toroidal pores.

In sum, the HS-AFM made possible the first molecular-level visualisation of the structures and dynamics of the AMP activity on membranes from its initial to final stages under infection-like conditions. We were able to determine unexplored details of the multifaceted pathways of the AMP action correlated to their dependence on the concentration and the elapsed time. We confirmed or disconfirmed so far hypothetical models and detected unknown molecular mechanisms and quantifications of interaction energies out of the reach of other techniques, including computer simulations^48,49^. Finally, biological and medical relevance of the approach was enhanced by the correlation of the molecular AFM information with information from SEM, TEM and OM on *B. subtilis* WT and mutant cells and non-supported POPG vesicles.

### Resistance to Dap by an increase of the CL membrane content

As already mentioned, bacterial resistance to Dap is increasing. In some strains, of *Enterococcus faecalis*, bacterial resistance to Dap has been directly related to increased content of cardiolipin in the membrane^63,64^. To investigate the mechanism of bacterial resistance, a model membrane composed of 20% molar TOCL (CL) 18:1 (1’,3’-bis[1,2-dioleoyl-sn-glycerol-3-phospho]-sn-glycerol) and 80% molar POPG was exposed to Dap. CL and POPG have similar alcyl chains and are mutually soluble^79^.

**Fig. 7:**
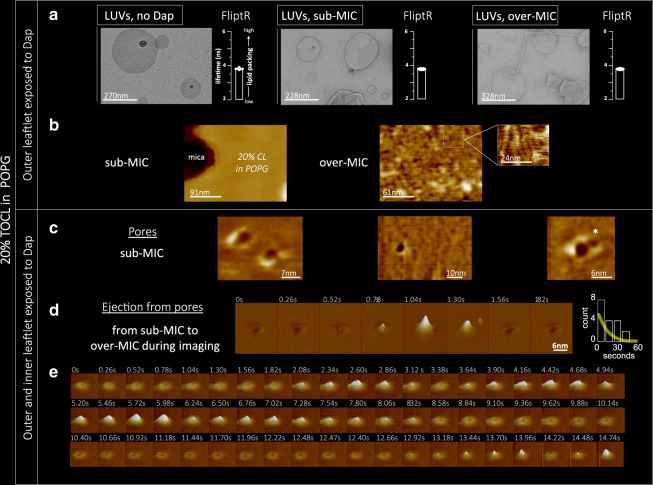
Dap effect on TOCL(CL)/POPG vesicles and model membrane by TEM, FliptR and HS-AFM. The incorporation of 20% molar of TOCL to the POPG model membrane inhibited Dap action creating CL-specific membrane dynamics. **a** By TEM, the TOCL/POPG vesicles do not deform at sub-MIC, whereas at over-MIC only minor deformations, scarce tubulations, are formed. By FliptR lifetime (standard deviation error shown), the lipid packing is not altered by Dap. **b**, left, On TOCL/POPG supported membranes exposed (tens of minutes) on the outer leaflet to sub-MIC Dap (colour scale: 4 nm), deformations were not observed, but, at over-MIC (**b,** right), membrane buckling appeared; periodically distributed lines with a 5 nm pitch reminiscent of a ripple phase (colour scale: 3 nm). The buckling static allowed high-resolution imaging, its substructure was composed of particles of 1.5 nm in diameter; possibly the head groups of Dap and/or TOCL molecules. **c** TOCL/POPG supported membrane of symmetrized inter-leaflet Dap distribution; for it, the POPG vesicles opened and adsorbed onto the mica substrate in the presence of Dap in the solution, toroidal pores formed of structure similar to the toroidal pores formed on POPG membranes (colour scale: 2 nm). The toroidal pores in TOCL/POPG showed a tendency to cluster (asterisk). **d** We induced inter-leaflet de-symmetrization by exposing the outer leaflet to supplementary quantities of Dap added to the imaging solution. This induced the activation of a possible membrane tension relief mechanism that, by a cyclic accumulation process seems to eject material out of the membrane; some material appears next to the pore at 1.30 s (Supplementary Movie 3) (Full colour scale: 3 nm). **d**, right, Histogram of the time-lapses between consecutive accumulations, the time variability spans from hundreds of milliseconds to several seconds, signature of the stochastic nature of the accumulation process. A fitting to an exponential curve is shown. **e** The pore recovers its initial state without any visible traces remaining from the material accumulation. Moreover, the symmetrization of Dap inter-leaflet distribution and the probable ejection of material from the pore caused the disappearance of the bucking deformation from the TOCL/POPG membranes, indicative of a membrane tension relief (Full colour scale: 3 nm).

We exposed non-supported CL/POPG vesicles to Dap and characterised it by FliptR measurements. We found that when CL was present, the lipid packing remained constant under Dap exposure, and that it was concentration-independent in the range studied (Fig. 7a, histograms). 20% of CL content, then, sharply changes the response of the membrane with respect the observations for POPG membranes and WT *B. subtilis* where Dap caused a relevant alteration of the lipid packing measured by FliptR (Fig. 2). It is interesting to mention that the *B. subtilis* 168 strain that we used contains 2.3% of CL at the exponential growth phase^80^. TEM observations confirmed that the CL/POPG vesicles were not deformed by the sub-MIC concentrations of Dap (Fig. 7a, images). However, higher concentrations of Dap created some tubulations, these could be imaged by TEM (Fig. 7a, right) but were undetected by the FliptR averaged signal.

On supported CL/POPG membranes, in line with the results on non-supported CL/POPG vesicles, sub-MIC exposure did not cause any deformations visible by AFM, neither dimples, toroidal pores nor tubules were observed (Fig. 7b) after tens of minutes of exposure. At higher concentration, instead, a high degree of static buckling appeared (Fig. 7b, right). At higher magnification, periodically distributed lines with a 5 nm pitch, reminiscent of ripple phase or the elongated humps appeared (Fig. 7b, inset), the lines displayed a periodically distribution (1.4 nm spacing) indicating a substructure of particles, probably constituted by head groups of Dap considering their dimensions^81,82^.

Overall, the AFM data confirm that higher contents of CL in the membrane strongly diminish the capability of Dap to perturb the POPG membrane. It has previously been suggested that the extra volume of the acyl-chain of CL would better accommodate Dap in the hydrophobic core of the membrane, avoiding the formation of energetically unfavourable voids, and thus, reducing curvature induction by Dap^30^. Our observations are certainly consistent with the idea that CL results in less deformation of the bilayer. However, it is surprising if Dap inserts stably into the bilayer that it does not change lipid packing as observed by FliptR, although it can agree with the observation of a CL-related stabilisation of membrane fluidity as observed on *Pseudomonas putida* exposed to surfactants^83^.

### Imposed symmetrization and de-symmetrization of the inter-leaflet distribution of Dap in a CL/POPG membrane

The previously described observation of a lack of toroidal pore formation on CL/POPG supported membranes exposed to Dap is coherent with previous findings (on CL/PG/PC bilayers) that show that CL inhibits Dap membrane permeabilization^25,60^. Such event has been hypothesised could derive from the blockage of the inter-leaflet translocation of Dap by the presence of CL in the membrane^25^. To investigate this hypothesis, a control experiment with a symmetric inter-leaflet distribution of Dap was devised. We symmetrized the inter-leaflet distribution of Dap, both in supported POPG and CL/POPG membranes, by incubating the lipid vesicles on the mica substrate in the presence of Dap in the incubation solution. As a result, during the opening of the vesicles on the mica substrate for the formation of the supported lipid bilayer, both leaflets of the membrane became exposed to the Dap, allowing the molecules to reach both leaflets prior to the adsorption of the bilayer on the mica support.

Applying this procedure with low Dap concentrations in solution, toroidal pores formed both in the POPG and the CL/POPG supported membranes. In the POPG membranes, the toroidal pores that formed (Supplementary Fig. 5) were indistinguishable from the toroidal pores forming in POPG membranes exposed to Dap after attachment (Fig. 5). In CL/POPG membranes, the pores formed by this procedure were very similar to those observed in POPG membranes, yet, in average, and besides their structural variability and their deviation from an ideal toroidal geometry, their size was slightly smaller than that of the pores in the POPG membranes (Fig. 7c and Supplementary Fig. 2). In the supported CL/POPG membranes, as in the supported POPG membranes, the toroidal pores were immobile. Nevertheless, they showed some tendency for clustering (Fig. 7c), although it was significantly smaller than that of the dimples (Fig. 4d), their clustering probably took place before the adsorption of the bilayers on the mica. Thanks to our control experiments we could confirm that, as was hypothesised in the literature, the CL incorporation blocks the formation of toroidal pores by impeding the translocation of Dap from the outer to the inner leaflet, and, thus, we support the model of Zhang et al.^25^ given that we proved that the lack of dimples stops the translocation of Dap molecules from the outer to the inner leaflet of the membrane; which leads us to reason that the dimples reduction of the membrane thickness increases the probability of Dap translocation.

As the symmetrization of the Dap distribution caused the appearance of pores, a subsequent de-symmetrization could induce other effects. We therefore induced inter-leaflet de-symmetrization by exposing the outer leaflet of the symmetrized CL/POPG membrane to extra Dap after the bilayers where adsorbed on the mica surface.

When the concentration of Dap in the imaging solution was increased to over-MIC the toroidal pores responded dramatically and dynamically. The HS-AFM imaging permitted us (Supplementary movie 4) to image a cyclic process occurring in the pores that consisted of three steps: Step 1: material, of undefined nature, accumulated at the centre of the pores (Fig. 7d, time 0.52 s). Step 2: the accumulation grew until it reached a diameter similar to the diameter of the pore, our AFM measurement (it applies tens to hundreds of piconewton forces) records a maximal height of ~3 nm that could be higher in reality (Fig. 7d, time 1.04 s). Step 3: the accumulated material in the pore disappeared, (it could be due either to the ejection of the material or to the re-insertion of the material in the membrane, the former seems more plausible provided the flux of Dap molecules from the solution inserted into the outer leaflet, plus the observation of material that appears next to the pore at the end of the accumulation process at the time of 1.30 s). In addition, the pore recovered its initial state without any visible traces remaining from the material ejection process (Fig. 7d, time 1.56 s). The process repeated with a new accumulation starting to grow at the centre of the pore (Fig. 7e), the time-lapses between the accumulations were irregular and the duration of the different cycles ranged from hundreds of milliseconds to several seconds (Fig. 7d), signature of a stochastic process (Fig. 7d right, the histogram of the time-lapses was fitted to an exponential). Moreover, the symmetrization of Dap inter-leaflet distribution and the ejection of material from the pores caused that at over-MIC the bucking deformation did not appear on the CL/POPG membranes (Fig. 7d).

## Discussion

A persistent problem in the studies of membrane-active antimicrobial peptides is the lack of experimental^6,7^ and in-silico^48,49^ methods for investigating their molecular configurations and processes in the membranes^29^. The leitmotif for this work was to develop new tools and protocols capable of scrutinising with molecular detail the multifaceted actions of antimicrobial lipopeptide molecules. There is a high intrinsic complexity in lipopeptide-membrane interactions that renders the design and development of lipopeptides antibiotics highly costly. At the same time, the complexity of the multifaceted actions of lipopeptides, and their easy access to their predominant target, the bacterial plasma membrane, renders the lipopeptides highly attractive candidates for the research of new antibiotic molecules. New advanced computer-assisted design strategies^45^ and new synthetic lipopeptides^11^ are improving the cost-effectiveness and the spectrum of potential lipopeptides. We focused on Dap, one of the few antibiotics that has achieved commercialisation in the last two decades for the treatment of Gram+ infections of which the details of its mode of action remains, as for any other lipopeptide, elusive.

**Fig. 8:**
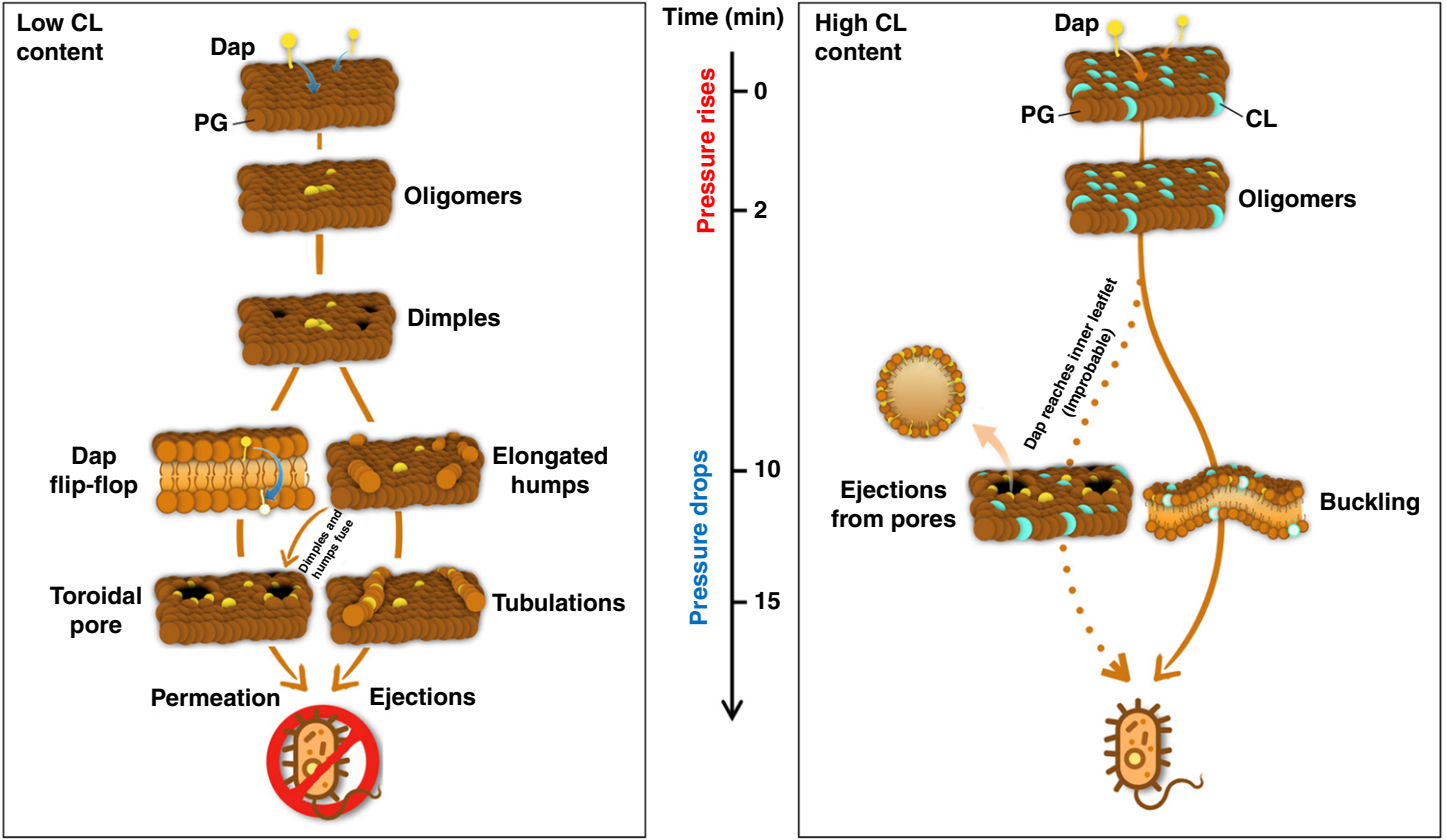
Schematics of the action pathways of Dap on Gram+ membranes. The timescale is indicated. The insertion of Dap molecules in the membrane rises the pressure. The pressure relief drives Dap actions: Left, When the membrane does not contain CL, the formation of Dap oligomers is followed by the formation of dimples in the membrane, followed by the formation of elongated-humps. The dimples favour the translocation of Dap to the inner membrane leaflet by the flip-flop mechanism. When Dap is present in the inner and outer leaflets toroidal pores form, the membrane is permeated, and the bacteria die. Alternatively, Dap can also induce ejection of material from the membrane by the formation of tubulations; equally destabilising the membrane and killing the bacteria. Right, Concerning the bacteria containing a high content of CL in the membrane (>20% molar), Dap action is drastically modified. In this case, the Dap flip-flop does not take place and the dimples are not formed. Instead, the membrane buckles but it does not break. Hence, despite Dap insertion, the membrane still separates the cytoplasm from the exterior. In the improbable case that some Dap molecules reach the inner leaflet of the membrane, pores form as in the case CL is present. The particularity of the pores formed under high CL content is that they are able of ejecting material out of the membrane. These pores behave ‘like geysers’, and relief the pressure built on the outer leaflet by the insertions of Dap molecules in cycles of activity. Both CL-related processes maintain the membrane integrity and the bacteria alive.

The HS-AFM imaging correlated with EM and OM enabled unprecedented assessment of structures and dynamics of the activity Dap on the membranes of living bacteria and mimic bilayers. We provided visual proof of previously hypothetical models, quantified interactions, and found unexpected new phenomena. Based on our findings, we discuss next our view on the activity of Dap on membranes (schematized in Fig. 8).

When Dap is added to POPG bilayers it inserts into the exposed monolayer thanks to its hydrophobic tail. This insertion results in an increase in the lateral pressure, as the area of the monolayer tries to increase but is retained by the geometry of the other monolayer. The inserted Dap molecules can form small dynamic aggregates in the exposed monolayer, as we have observed. Nevertheless, the system would like to reduce the lateral pressure: we found two different pathways of action for this. In a first mechanism the lateral pressure is reduced by flip-flop with Dap molecules passing to the second, unexposed, monolayer. This pathway leads to the formation of toroidal pores and is associated with killing by membrane permeation. In a second mechanism, the exposed monolayer can extrude POPG/Dap tubules with a relatively high Dap composition. These tubules are somewhat reminiscent of cylindrical mixed micelles and this pathway is associated with killing by lipid loss.

In the context of this model for Dap action we would suggest that the flip-flop mechanism requires a relatively low Dap concentration in the outer-leaflet, as when it is over-occupied the lateral pressure prevents the passage of the large head-group into the membrane. This is consistent with the role proposed for the dimples, that we have observed, in catalysing inter-leaflet transfer, as this structure also increases monolayer surface area or lateral pressure. We suggest that at relatively high surface densities Dap aggregates are able to reduce the lateral pressure by forming elongated-humps in the membrane. Such humps can evolve in several ways. Indeed, they can aggregate to form the ripple like phase that we observed, they can fuse with a dimple pushing Dap to the other side of the membrane in the process or they can develop into tubules.

The model also allows a coherent vision of the different dynamical processes that we have observed. The membrane leaflet exposed to high Dap concentrations rapidly accumulates large amounts of Dap and the lateral pressure rises. This initial unstable system relaxes, over a period of hours, as the lateral pressure drops and tubules form. Toroidal pores are prevented from formation by the absence of dimples that catalyses inter-leaflet transport of Dap. Equally, the membrane containing pores exposed to extra Dap produces (under the presence of CL) not tubes but objects escaping from toroidal pores. These, we believe to be Dap rich mixed micelles whose production is catalysed by the pores. This process is powered by the relief of the accumulated pressure caused by the insertions of Dap and reminiscent of the process of relief of accumulated pressure of geysers.

We expect that the potential of the HS-AFM imaging will stimulate new models and insight on the structure-activity relationship of membrane-interacting molecules. For example, U.S. Food and Drug Administration recently approved antibiotics with similarities with Dap (oritavancin, dalbavancin, and telavancin), also medically used for complicated skin infections by Gram+ bacteria. These molecules, such as Dap, anchor the bacterial membrane by a lipophilic side chains, but the molecular mechanisms involved in membrane rupture are undetermined; they may involve either the formation of membrane pores or the lysis of the membrane^84^, Hence, for the pharmaceutical industry, HS-AFM label-free assessment that does not require biochemical synthesis and provides novel information, represents an opportunity to considerably increase the throughput of screening of molecular candidates. In addition, it also permits to better point the molecular candidates based on a more complete understanding of the structure-activity relationships.

## Methods

### Non-supported-model membrane experiments

Large unilamellar (LUVs) and giant unilamellar (GUVs) vesicles were used for TEM and FLIM assays, respectively.

LUVs were prepared by evaporation of chloroform, rehydration of the lipid solution, and freeze-thawed cycles. Next, the vesicles were incubated with Dap. Finally, the solution was deposited on a glow-discharged carbon-coated EM grid and let to adsorb. The sample was negatively stained with 2% uranyl acetate and imaged by TEM.

GUVs were prepared by a modified protocol of the electro-formation technique^85^ in the presence of FliptR, exposed to a given concentration of Dap and imaged on a flow chamber by FLIM technique.b

### Protein

One milligram of Daptomycin (Dap) powder (D2446-1MG, Sigma-Aldrich, Darmstadt, Germany) was dissolved in 1 ml of sterilised water to prepare the stock solution (final concentration 1 mg/ml). Aliquots of 20 µl of the stock were kept at −20 °C until use. The stock solution was thawed at room temperature, diluted in sterilised water to obtain the desired concentrations (20 and 0.6 μM) for the experiment and stored at 4 °C for one week.

### Lipid

Stocks of lipids POPG 16:0-18:1 (1-palmitoyl-2-oleoyl-*sn*-glycero-3-phospho-(1’-*rac*-glycerol), 840457C-25 mg, Avanti Polar Lipids, Alabama, USA) and Cardiolipin (TOCL) 18:1 (1’,3’-bis[1,2-dioleoyl-*sn*-glycero-3-phospho]-*sn*-glycerol, 710335C-25 mg, Avanti Polar Lipids, Alabama, USA) were kept in chloroform at −20 °C until use.

### Minimal Inhibition Concentration assay (MIC)

*B. subtilis* WT^65^, *ugtP*^66^ *and mreB*^68^ mutant strains were grown in LB (Luria-Bertani) liquid medium supplemented with 10 mM MgSO_4_ and 1.25 mM CaCl_2_ at 37 °C and 225 revolutions per minute to early log phase (OD_530_, 0.3). A 0.1 ml of the culture were incubated for 5 h at 37 °C into a 96-well titre plate containing 0.1 ml of LB medium supplemented with 10 mM MgSO_4_ and 1.25 mM CaCl_2_ with serially diluted concentrations of Dap (from 6 nM up to 6 µM). Bacterial growth at various Dap concentrations was tracked by measuring the OD_540_ after 5 h. It was expressed as the percent increase compared to the observed OD_540_ in the absence of Dap. The relative resistance of mutant strains was calculated using the ratio between the drug concentration that gave 50% inhibition of growth and the one required to inhibit growth by 50% in the WT.

### TEM

Lipid stock solutions were thawed at room temperature, mixed in 2 ml microfuge tubes to obtain the desired molar ratios (POPG and POPG:TOCL 4:1, mol:mol) and achieve a final concentration of 1 mg/ml in both. To form large unilamellar vesicles (LUVs), the chloroform was evaporated under a gentle flow of nitrogen, followed by 30 min incubation in a vacuum oven at 30 °C. Hereafter, lipids were fully rehydrated with the “Hydration buffer” (10 mM HEPES (4-(2-hydroxyethyl)-1-piperazineethanesulfonic acid), 100 mM NaCl, pH 7.4) for 10 min at room temperature. Finally, the LUVs suspension was vortexed for 10 s and freeze-thawed three times in liquid nitrogen and a water bath, respectively. The final LUVs solution was incubated for 10 min at room temperature with the desired concentration of Dap (20 and 0.6 μM), in the presence of 1.25 mM Ca^2+^. A drop of the solution was deposited on a glow-discharged carbon-coated EM grid and left to adsorb. Finally, the grid was blotted with a filter paper and negatively stained with 2% uranyl acetate for 30 s for TEM analysis (FEI Tecnai G2 Sphera electron microscope, FEI Company, Oregon, USA).

Likewise, to form a homogeneous SLB, the LUVs suspension was incubated overnight at room temperature on a glow-discharged carbon-coated EM grid. After SLB formation, the sample was carefully rinsed with Guanosine Triphosphate (GTP) buffer. To test the Dap effect, the SLB was incubated for 10 min at room temperature with the desired Dap concentrations (20 and 0.6 μM), in the presence of 1.25 mM Ca^2+^. Finally, the grid was blotted with a filter paper and negatively stained with 2% uranyl acetate for 30 s for TEM analysis.

### Supported lipid bilayer preparation for HS-AFM

The lipid stock solutions were thawed at room temperature and successively mixed in 2 ml microfuge tubes to obtain the desired molar ratios (POPG and POPG:CL 4:1, mol:mol) and achieve a final concentration of 1 mg/ml in both. The chloroform was evaporated under a gentle flow of nitrogen. Then, the samples were kept overnight under reduced pressure. The samples were rehydrated with the “Hydration buffer” heated at 65 °C. Next, they were immersed in a thermal bath at 65 °C for 45 s followed by 45 s of vortexing. The heating-vortexing cycle was repeated 5 times. Finally, the samples were sonicated for 30 min in an ultrasonic bath (*f* ~ 35 kHz) to obtain the liposome solutions. The solutions were stored at 4 °C until use. To form a homogeneous supported lipid bilayer (SLB), a mixture of 100 µl of the liposome suspension (1 mg/ml) and 100 µl of “Incubation buffer” (10 mM HEPES pH 7.4, 100 mM NaCl, 5 mM CaCl_2_) was incubated overnight at room temperature on a freshly cleaved mica under a humid hood. The sample was rinsed 10 times in ultrapure water (MilliQ) and imaged by HS-AFM in “Imaging buffer” (10 mM HEPES pH 7.4, 100 mM NaCl, 1.25 mM CaCl_2_) at 37 °C to check the homogeneity of the formed SLB prior to Dap incubation.

### ‘Fault current detection’ method for the measurement of the cantilever oscillation

In collaboration with Hinstra Instruments Ltd., Kalvaria sgt. 53-55/B/C, H6725 Szeged, Hungary. A detection of the amplitude on 1/2 cycle of oscillation was adapted for the HS-AFM technology based on the ‘fault current detection’ method; commonly used at the electrical distribution industry^86^, not yet at the AFM technology. For the implementation of the ‘fault current detection’ method, we used a Lock-in with a reconfigurable FPGA that allowed us to sample each half-period of oscillation by 75–100 datapoints; a sufficient level of oversampling to average the signal and considerable reduce the noise of reading. Moreover, to obtain precise amplitude readout, prior to the measurement, we used a high pass filter to remove the DC level.

We show the algorithm of the ‘fault current detection’ below in its discrete (time/sample dependent) form for a digitalised signal given by ${{x}}_{{i}} = {{A}} \cdot \sin \left( {\frac{{2\pi \cdot {{i}}}}{{{N}}} + \varphi } \right)$, where *A* is the amplitude, *φ* is the phase, and *x*_*i*_ is the ith measurement of the sinusoidal input which has a period of *N* samples:

1 $$A = \frac{4}{N}\sqrt {\left( {\mathop {\sum}\nolimits_{i = 0}^{\frac{N}{2} - 1} {x_i} \cdot {\mathrm{cos}}\left( {\frac{{2\pi \cdot i}}{N}} \right)} \right)^2 + \left( {\mathop {\sum}\nolimits_{i = 0}^{\frac{N}{2} - 1} {x_i} \cdot \sin \left( {\frac{{2\pi \cdot i}}{N}} \right)} \right)^2}$$

### HS-AFM imaging

HS-AFM movies were performed in amplitude modulation mode optimised high–resolution imaging parameters with a modified HS-AFM (SS-NEX, RIBM, Tsukuba, Japan)^87^. The apparatus is equipped with our custom-made digital high-speed lock-in amplifier coded on a reconfigurable FPGA using LabView (National Instruments, Austin, USA). Short cantilevers (length ~7 µm) designed for HS–AFM, presenting an electron beam deposition (EBD) tip, were used (USC-F1.2-k0.15 Nanoword, Neuchâtel, Switzerland). They are characterised by a nominal spring constant *k* = 0.15 N/m, a resonance frequency in liquid of *f*_res_ = 500 kHz and a quality factor *Q*_c_ ~ 2 (in liquid). HS-AFM sensitivity to probe the deflection was 0.1 V/nm. The imaging amplitude setpoint was set to ~90% of the free amplitude (~10 Å). All experiments were performed in “Imaging buffer” at 37 °C, by introducing the HS-AFM microscope inside a thermo-controlled box.

### HS-AFM image treatment

Image treatment was limited to the correction of XY drift and a first order X-line fit. Image analysis was performed using the general distribution of ImageJ^88^ and of WSxM^89^.

### SEM experiment

SEM technique allows the direct visualisation of the morphological changes of the bacteria upon Dap incubation. Gram-positive *B. subtilis* wild-type, *mreB* and *ugtP* mutants were grown in LB medium in the presence of 25 mM MgSO_4_ at 37 °C and 216 × *g* to early log phase (OD_600_, 0.3).

Each culture was divided into three samples of 700 µl to test different Dap incubation conditions, namely:

1. No Dap, 1.25 mM Ca^2+^ (negative control);
2. 0.6 µM Dap, 1.25 mM Ca^2+^, 1 h at 37 °C;
3. 20 µM Dap, 1.25 mM Ca^2+^, 1 h at 37 °C;

Then, the samples were washed with 0.1 M cacodylate buffer (Na(CH_3_)_2_ AsO_2_ · 3H_2_O, pH 7.2) and fixed with 2.5% glutaraldehyde for 20 min at room temperature. The samples were centrifuged (5 min, 3840 × *g*), and afterward, the supernatant was removed. The pellet was washed with cacodylate buffer 0.05 M, centrifuged 5 min at 3840 × *g* and suspended in Millipore water (MilliQ). After a further centrifugation step, the sample was stored at 4 °C in MilliQ overnight. Next, bacteria were collected on a filter and dehydrated by exposure to a series of ethanol washing (25%, 50%, 80%, 100% twice). Finally, each filter was immersed in HMDS (hexamethyldisilazane) twice, and the residues of the solvent were let evaporate under the hood. The samples were coated with gold for 2 min and imaged by SEM (FEI TENEO VS microscope, FEI Company, Oregon, USA).

### Fluorescence light microscopy (optical microscopy)

*B*. *subtilis* wild-type, *mreB* and *ugtP* mutants were grown in LB medium (for *mreB* 25 mM MgSO4 were added to the medium) at 37 °C and 225 revolutions per minute to early log phase (OD_600_ = 0.3). 1 ml of each solution was used as negative control and directly marked with the lipophilic membrane probe FM1-43 dye ((N-(3-Triethylammoniumpropyl)-4-(4-(Dibutylamino) Styryl) Pyridinium Dibromide), T3163, Thermo Fisher Scientific, Waltham, USA), which inserts into the outer leaflet of the cell membrane and becomes fluorescent. The remaining culture solution was divided into two tubes, and either 1.25 mMCa^2+^ or 1.25 mMCa^2+^, 20 µM Dap were added to the solution. The samples were incubated for 1 h at 37 °C. Next, the samples were marked with the FM1-43 dye and observed with fluorescence light microscopy. The experiment was repeated with a lower concentration of Dap (0.6 µM).

### FliptR FLIM on GUVs and bacteria

Giant unilamellar vesicles (GUVs) were prepared using a modified protocol of the electroformation technique^85^. The lipid solution was supplemented with FliptR (from DMSO solution) at 1% mol and 0.03% mol DSPE-PEG2000-Biotin (1,2-distearoyl-sn-glycero-3-phosphoethanolamine-N-[biotinyl(polyethylene glycol)-2000], Avanti Polar Lipids, Alabama, USA). FliptR is a membrane mechanosensitive probe used to identify changes in lipid packing^57^. The lipid solution was spread on ITO-coated glass slides, previously cleaned with bi-distilled water (ddH_2_O), ethanol and chloroform. The slides were kept for 2 h at 37 °C to allow drying of the lipid solution. A chamber was assembled using the ITO-coated glass slides separated by an O-ring. The chamber was filled with a 297 mOsm sucrose solution and blocked with silicone elastomer. An electric field (10 Hz, 1.1 V) was then applied for 2 h at 55 °C. Lifetime imaging of GUVs was performed in a flow chamber. Prior to addition of the GUVs solution, the coverslip was assembled to form the flow chamber (sticky-Slide VI 0.4, Ibidi, Munich, Germany) and incubated with 0.1 mg/ml avidin (Avidin from egg white, 434401, Life Technologies, Thermo Fisher Scientific, Waltham, USA) for 10 min. Avidin was washed out 3 times with ddH_2_O, and the flow chamber was filled with 200 μl of PBS. 5 μl of GUVs were then flushed inside the flow chamber. Once the GUVs started to partially adhere to the surface and to avoid further adhesion, which could lead to GUV bursting, the remaining glass-bound avidin was blocked by flowing BSA-biotin (1 mg/ml, A8549, Sigma-Aldrich, Darmstadt, Germany) during 15 min.

Bacteria cells were growth as previously described for SEM measurements. Upon 1 h Dap incubation (0.6 μM and 20 μM), the bacteria were incubated with 2 μM of FliptR for 30 min and afterward injected into the flow chamber previously coated with 1 mM of poly-L-lysin for 10 min and washed 3 times.

FLIM imaging was performed using a Nikon Eclipse Ti A1R microscope (Nikon Corporation, Tokyo, Japan) equipped with a Time-Correlated Single-Photon Counting module from PicoQuant^90^. Excitation was performed using a pulsed 485 nm laser (LDH-D-C-485, PicoQuant, Berlin, Germany) operating at 20 MHz, and the emission signal was collected through a bandpass 600/50 nm filter using a gated PMA hybrid 40 detector and a TimeHarp 260 PICO board (PicoQuant, Berlin, Germany).

### Analysis of fluorescence decay data

SymPhoTime 64 software (PicoQuant, Berlin, Germany) was used to fit fluorescence decay data (from full images or regions of interest) to a dual exponential model for GUVs measurements and 3 exponential model for bacteria, after deconvolution for the instrument response function (measured using the backscattered emission light from a solution of 1 μM of fluorescein and 4 M of KI). Data were expressed as (mean ± standard deviation of the mean). The full width at half-maximum (FWHM) response of the instrument was measured at 176 ps.

## Supplementary information

Supplementary Information

Description of Additional Supplementary Files

Supplementary Movie 1

Supplementary Movie 2

Supplementary Movie 3

Supplementary Movie 4

## Data Availability

The data that support the findings of this study are available from the corresponding author upon reasonable request.
