# Supplementary material for: High-speed atomic force microscopy highlights new molecular mechanism of daptomycin action
Source: Nat Commun. 2020 Dec 9;11:6312. doi: 10.1038/s41467-020-19710-z (PMC7725780; doi:10.1038/s41467-020-19710-z)
Supplement: Supplementary file 1 — Supplementary Information [file 41467_2020_19710_MOESM1_ESM.pdf]

Supplementary information

Mode of action of daptomycin: Evidence of new molecular mechanisms highlighted by high-speed atomic force microscopy

Francesca Zuttion[1], Adai Colom[2], Stefan Matile[3], Denes Farago[4] , Frédérique Pompeo[5], Janos Kokavecz[6], Anne Galinier[5], James Sturgis[7], Ignacio Casuso[1*]

[1] U1067 INSERM, Aix-Marseille Université, Marseille, France

[2] Biochemistry Department, University of Geneva, Geneva, Switzerland

[3] Organic Chemistry Department, University of Geneva, Geneva, Switzerland

[4] Department of Technical Informatics University of Szeged, Szeged, Hungary

[5] Laboratoire de Chimie Bactérienne (LCB), Institut de Microbiologie de la Méditerranée (IMM), CNRS, UMR 7283, Aix Marseille Université, Marseille, France

[6] Institute of Environmental Science and Engineering, University of Szeged, Szeged, Hungary

[7] LISM, UMR 7255, CNRS, Aix Marseille Université, Marseille, France

* email: ignacio.casuso@inserm.fr

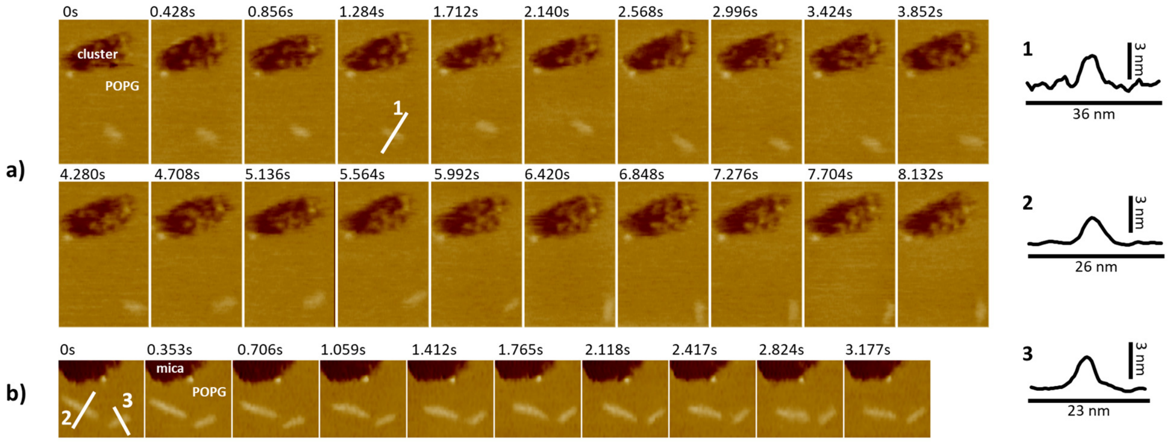

Supplementary Fig. 1. Elongated-humps on the POPG bilayer after a few tens of minutes of exposure to sub-MIC Dap. (a) Motion of one elongated-humps close to a cluster of dimples, and corresponding height profile (colour scale: 7nm). (b) Motion of two elongated-humps close to the border of the POPG patch, and corresponding height profiles (colour scale: 7nm).

a) b)

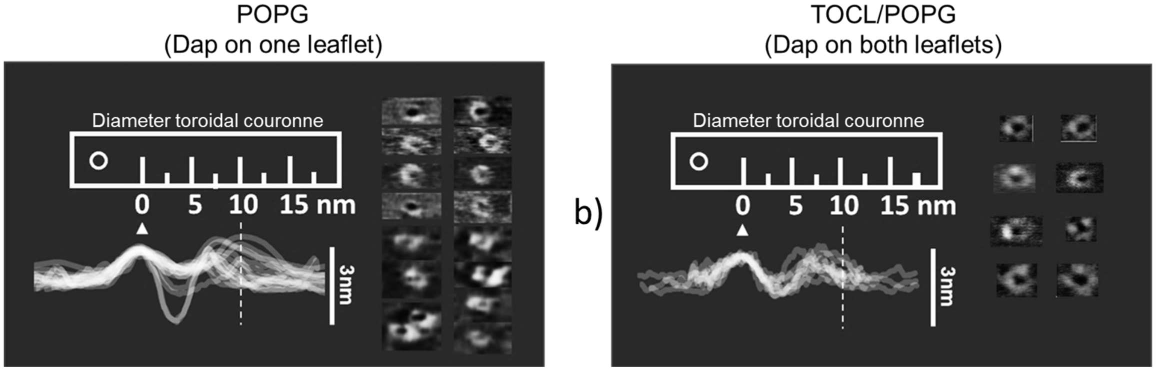

Supplementary Fig. 2. AFM topographies of the pores that Dap induces on the supported membranes. Because our HS-AFM tips do not fit into the pores and the structure of the pores deviates from the ideal toroidal shape, the assessment of the pore diameter is, to some extent, imprecise. We show the visual comparative of the profiles of the pores (a) on POPG bilayers —only one leaflet was exposed to Dap—, the dispersion of the diameter of the toroidal couronnes (peak-to-peak) ranges from 6 to 11 nm. (b) On TOCL/POPG bilayers —we exposed in this case both leaflets to Dap—, the visual comparative of the profiles of the pores shows a dispersion of the toroidal couronnes (peak-to-peak) from 5 to 8 nm.

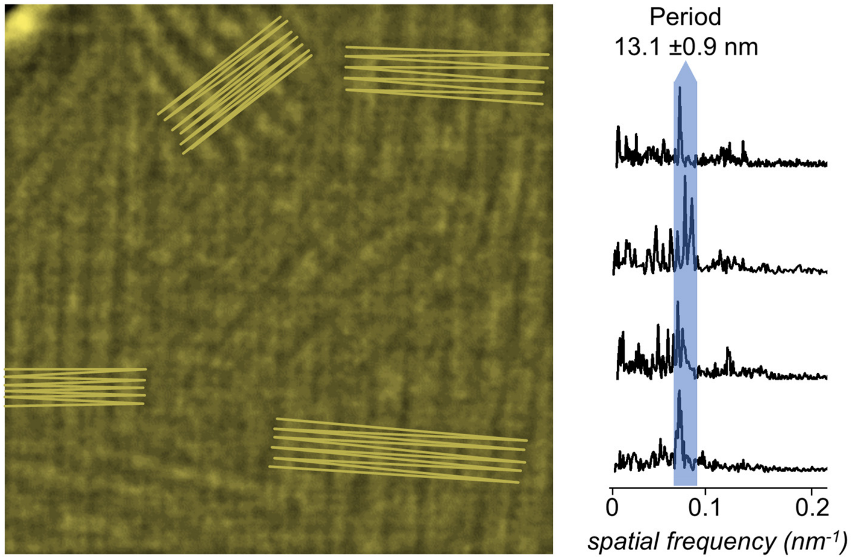

Supplementary Fig. 3. Periodicity of the ripple phase domains that arise when supported POPG bilayers are exposed to over-MIC Dap. (a) The zones of ripple phase can show domains with different orientations (colour scale: 3nm). **(b)** Spatial frequency (by Fourier Transform) of the topographic profiles shown in (a) that attest that the pitch of the ripples is largely conserved among domains; with a mean and the standard deviation of 13.1 ± 0.9.

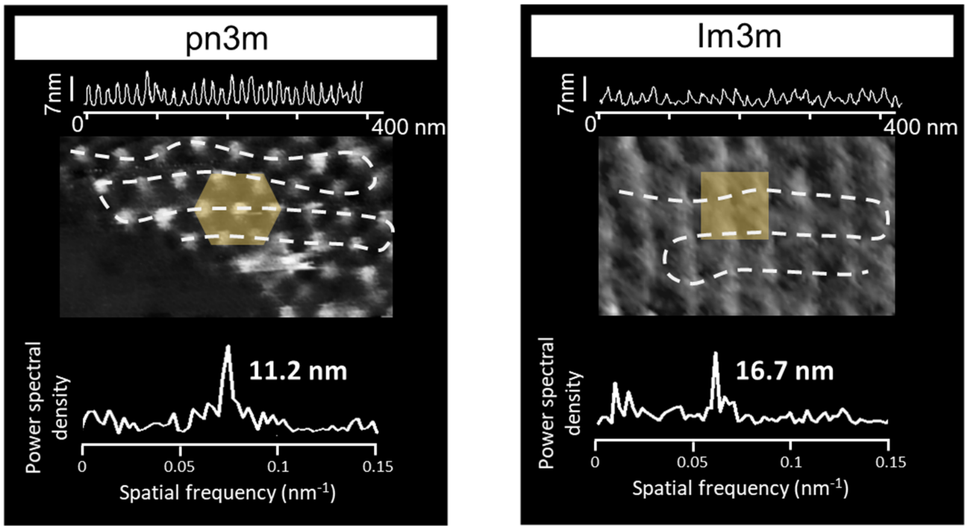

Supplementary Fig. 4. Periodicity of the Pn3m and lm3m cubic phases created on the POPG supported bilayer by exposure to over-MIC Dap. The yellowish hexagon and square show the respective unit cells, the perimeter, and the side lengths of the Pn3m and lm3m cubic phases. The topographic profiles across the highest points of the cubic phases (slashed lines and plots on top of the images) are used to derive the side length of the cubic phases: for the Pn3m hexagonal lattice is 11.2 nm, and for the lm3m square lattice is 16.7 nm.

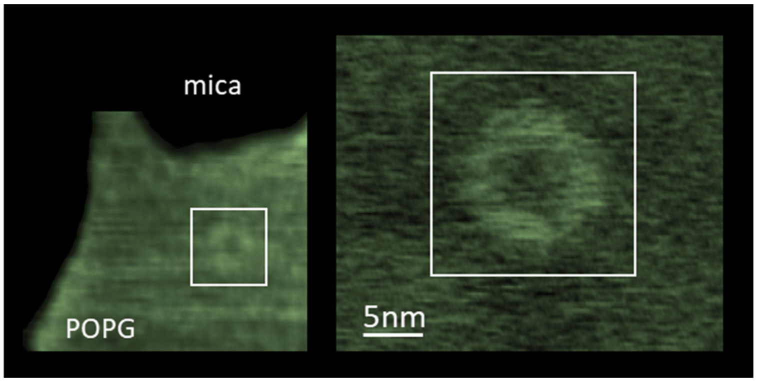

Supplementary Fig. 5. Toroidal pore formed on a POPG bilayer by symmetrized exposure to Dap (colour scale: left: 4nm, right: 3nm). The shape and dimensions is similar to the toroidal pores formed by single side exposure of the POPG bilayer to Dap.
