# Supplementary material for: High-speed atomic force microscopy highlights new molecular mechanism of daptomycin action
Source: Nat Commun. 2020 Dec 9;11:6312. doi: 10.1038/s41467-020-19710-z (PMC7725780; doi:10.1038/s41467-020-19710-z)
Supplement: Supplementary file 2 — Description of Additional Supplementary Files [file 41467_2020_19710_MOESM2_ESM.pdf]

Description of Additional Supplementary Files in:

Mode of action of daptomycin: Evidence of new molecular mechanisms highlighted by high-speed atomic force microscopy

Francesca Zuttion[1], Adai Colom[2], Stefan Matile[3], Denes Farago[4] , Frédérique Pompeo[5], Janos Kokavecz[6], Anne Galinier[5], James Sturgis[7], Ignacio Casuso[1*]

[1] U1067 INSERM, Aix-Marseille Université, Marseille, France

[2] Biochemistry Department, University of Geneva, Geneva, Switzerland

[3] Organic Chemistry Department, University of Geneva, Geneva, Switzerland

[4] Department of Technical Informatics University of Szeged, Szeged, Hungary

[5] Laboratoire de Chimie Bactérienne (LCB), Institut de Microbiologie de la Méditerranée (IMM), CNRS, UMR 7283, Aix Marseille Université, Marseille, France

[6] Institute of Environmental Science and Engineering, University of Szeged, Szeged, Hungary

[7] LISM, UMR 7255, CNRS, Aix Marseille Université, Marseille, France

* email : ignacio.casuso@inserm.fr

Supplementary. Movie 1

HS-AFM movie of the first minutes after exposure to sub-MIC Dap on a POPG supported membrane. Guides to the eye highlights those oligomers identifiable.
Movie parameters: frame rate 33 ms; full image of 90 nm x 65 nm and 256x180 pixels; colour depth 8bit (256 values); full colour scale 4 nm

Supplementary. Movie 2

HS-AFM movie after tens of minutes after exposure to sub-MIC Dap that shows diffusing dimples on a POPG supported membrane which interact by swinging trajectories.
Movie parameters: frame rate 83 ms; full image of 150nm x 150nm and 256x256 pixels; colour depth 8bit (256 values); full colour scale 4 nm

Supplementary. Movie 3

HS-AFM movie of the first minutes after exposure to over-MIC of a POPG supported membrane. A flow of material is visualized thanks to the motion of the ripples, it starts at the lm3m cubic phase (left) and ends at a tubulation (right).
Movie parameters: frame rate 456 ms; full image of 400nm x 400nm and 300x300 pixels; colour depth 8bit (256 values); full colour scale 16 nm

Supplementary. Movie 4

HS-AFM movie of the cyclic accumulation of material in the pores created on TOCL/POPG supported membranes under the exposure of the outer leaflet to supplementary quantities of Dap added to the imaging solution. The process seems to eject material out of the membrane; see the material that appears next to the pore at 1.30s.
Movie parameters: frame rate 260 ms; zoom of a full image of 140nm x 100nm and 256x180 pixels; colour depth 8bit (256 values); full colour scale 3 nm
